# The natural triterpene 3β,6β,16β-trihydroxy-lup-20(29)-ene obtained from the flowers of *Combretum leprosum* induces apoptosis in MCF-7 breast cancer cells

**DOI:** 10.1186/1472-6882-14-280

**Published:** 2014-08-02

**Authors:** Cassiana Macagnan Viau, Dinara Jaqueline Moura, Valdir Alves Facundo, Jenifer Saffi

**Affiliations:** Department of Basic Health Sciences, Laboratory of Genetic Toxicology - UFCSPA, Porto Alegre, RS Brazil; National Institute for Translational Research on Health and Environment in the Amazon Region - CNPq/INCT/INPeTAm, Rio de Janeiro, RJ Brazil; Department of Medicine, Federal University of Rondônia - UNIR, Porto Velho, RO Brazil

**Keywords:** *Combretum leprosum*, 3β, 6β, 16β-trihydroxy-lup-20(29)-ene triterpene, Antiproliferative activity, Cleaved caspase-9, ROS, Mitochondrial apoptotic pathway

## Abstract

**Background:**

The 3β, 6β, 16β-trihydroxylup-20(29)-ene (TTHL) is a pentacyclic triterpene obtained from the medicinal plant *Combretum leprosum* Mart. In folk medicine, this plant is popularly known as mofumbo, cipoaba or mufumbo, and is used to treat several diseases associated with inflammation and pain.

**Methods:**

We investigated the antitumor efficacy of TTHL isolated from *C. leprosum*. The TTHL cytotoxic effect was investigated in MRC5, MCF-7, HepG2, T24, HCT116, HT29, and CACO-2 cells after 24, 48, 72 and 120 h of treatment. The mechanisms of cell death and DNA damage induction were investigated by flow cytometry and comet assay, respectively.

**Results:**

The results indicated that TTHL induced a time- and concentration-dependent growth inhibition in all human cancer cell lines. The cytotoxicity was more pronounced in MCF-7 breast cancer cells, with an IC50 of 0.30 μg/mL at 120 h. We therefore evaluated the cell death mechanism induced by TTHL (IC20, IC50, and IC80) in MCF-7 cells at 24 h. We found that the treatment with IC50 and IC80 TTHL for 24 h induced apoptosis in 14% (IC50) and 52% (IC80) of MCF-7 cells. The apoptosis induced by TTHL was accompanied by increased levels of both cleaved caspase-9 and intracellular ROS. In order to further understand the biological mechanism of TTHL-induced cytotoxicity, we have also investigated its effect on different *Saccharomyces cerevisiae* yeast strains. The mutant strains *sod1*Δ, *sod2*Δ, and *sod1*Δ*sod2*Δ, which are deficient in superoxide dismutase antioxidant defenses, were hypersensitive to TTHL, suggesting that its capacity to disturb cellular redox balance plays a role in drug toxicity. Moreover, TTHL induced mutagenicity in the yeast strain XV185-14c.

**Conclusions:**

Taken together, the results suggest that TTHL forms covalent adducts with cellular macromolecules, potentially disrupting cellular function and triggering apoptosis.

**Electronic supplementary material:**

The online version of this article (doi:10.1186/1472-6882-14-280) contains supplementary material, which is available to authorized users.

## Background

Medicinal plants have been used since ancient times in virtually all cultures as a source of medicines [1], and are of great importance to the health of individuals and communities [2]. Traditional medicine is used in all parts of the World and has a rapidly growing economic importance, mainly through the use of medicinal plants, especially in developing countries [3]. The medicinal use of plants of the Combretaceae family is widely described in the scientific literature [4–6]. This family is distributed in 20 genera, with approximately 600 species. The largest genera are *Combretum* and *Terminalia*, with about 370 and 200 species, respectively [7]. Members of Combretaceae occur mainly in tropical and subtropical areas, such as Africa and Brazil [8].

**Figure 1:**
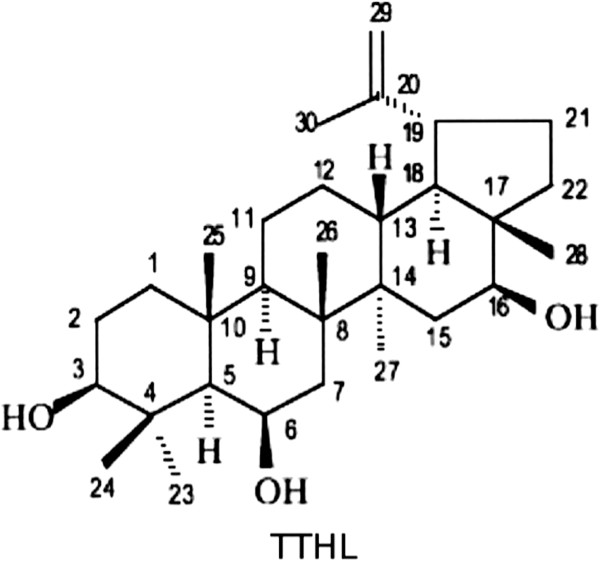
**Chemical structure of TTHL (3β,6β,16β-trihydroxy-lup-20(29)-ene) isolated product from** ***Combretum leprosum.***

In North and Northeastern Brazil, the species *Combretum leprosum* Mart. is popularly known as mofumbo, cipoaba or mufumbo. Infusions prepared with the aerial parts (stems, leaves and flowers) and roots of *C. leprosum* are used in folk medicine to heal wounds, to treat hemorrhages, or as a sedative [9, 10]. According to phytochemical analysis, *C. leprosum* is rich in compounds such as cycloartanes, triterpenes [arjunolic, mollic acid and 3β,6β,16β-trihydroxy-lup-20(29)-ene (TTHL - Figure 1)], and flavonoids (3-O-methylquercetin, 5,3′-dihydroxy-3,7,4′-trimethoxyflavone, 5,3′,4′-trihydroxy-3,7-dimethoxyflavone, and quercetin), and some of these substances have proven biological activity [9, 11–15].

The pentacyclic triterpene TTHL, obtained from the flowers of *C. leprosum* [16], presents a pronounced antinociceptive effect [11], which is dependent on opioid and serotonergic systems, G(i/o) protein activation and the opening of specific K(+) channels [11]. Behavioral and electrographic recordings revealed that pretreatment with TTHL increased the latencies to the first clonic seizure to the tonic-clonic and reduced the duration of the generalized seizures induced by the GABA(A) receptor antagonist pentylenetetrazol [17].

Although considerable work has been done on the plant for different activities, scientific information on toxicological properties of TTHL is still not available. Since elimination of cancer in early stages is an integral part of chemoprevention, the assessment of antiproliferative properties against cancer cells provides useful insights on the chemo-protective potential of natural products. In this sense, we evaluated the abilities of TTHL to function as an antitumoral drug. Moreover, to further understand the biological mechanism of the cytotoxic effect of TTHL, our group also investigated its effect on different *Saccharomyces cerevisiae* strains.

## Methods

### Chemicals

Dulbecco’s modified Eagle’s medium (DMEM), low-melting-point agarose (LMP), high-melting-point agarose (HMP), phosphate-buffered saline (PBS; Na_2_HPO_4_, KH_2_PO_4_ and KCl, pH 7.4), propidium iodide (PI), mitoxantrone (MXT), hydrogen peroxide (H_2_O_2_), amino acids and nitrogenated bases were purchased from Sigma (St. Louis, MO, USA). Fetal bovine serum (FBS) and penicillin/streptomycin were obtained from Gibco-BRL (Grand Island, NY, USA). Primary antibody anti-caspase-9 and secondary antibody anti-rabbit IgG (H + L) F(ab’)2 fragment conjugated to Alexa Fluor® 488 were obtained from Cell Signaling Technology (USA) and Invitrogen (Grand Island, NY, USA), respectively. Cell Proliferation Kit II (XTT) was acquired from Roche (Basel, Switzerland). Annexin V-Phycoerythrin (PE) and 7-Amino-Actinomycin (7-AAD) were purchased from BD Biosciences (San Diego, CA). Yeast extract, bacto-peptone, bacto-agar and yeast nitrogen base were obtained from Difco Laboratories (Detroit, MI). All other reagents were of analytical grade.

### Plant material and TTHL isolation

Botanical material was collected by Dr. Edilberto Rocha Silveira (Federal University of Ceará, Fortaleza) in May 2007 in a free area of Viçosa, Ceará State, Brazil, and classified by Dr. Afrânio Fernandes (Federal University of Ceará, Fortaleza) as *Combretum leprosum* Mart. A voucher specimen of this plant was deposited in Herbarium Prisco Bezerra of the Biology Department, Federal University of Ceará, Brazil, under number 12446. All necessary permits were obtained for the harvesting of the flowers.

The isolation of TTHL triterpene has been described by Facundo *et al.* [16]. Briefly, the dried flowers (2.7 kg) were powdered and extracted with ethanol (5 L), being stirred and macerated at room temperature (24 ± 3°C) for approximately 24 h. This procedure was repeated three times. The solvent was fully evaporated under reduced pressure and the EE (yield 58.3 g) was lyophilized and stored in a freezer at -20°C until use. Part of the EE (32.0 g) was subjected to column chromatography on silica gel, eluting with n-hexane, chloroform (CHCl_3_), ethyl acetate (EtOAc) and methanol (MeOH). The fraction eluted with CHCl_3_ was chromatographed on a silica gel column and was eluted with n-hexane-EtOAc, in increasing polarity. The fractions 27–30, eluted with n-hexane-EtOAc (30:70), were combined on the basis of thin layer chromatography (TLC) analysis, and the presence of a white precipitate was observed, which after recrystallization from ethanol was identified as TTHL (2.37 g). The analysis of ^1^H NMR and ^13^C NMR spectra showed that TTHL analytical and spectroscopic data fully agreed with their assigned structures [16], and the chemical purity of TTHL was > 98% [11, 12]. For cell treatments, a stock solution of the TTHL was prepared freshly prior to use, using dimethylsulfoxide (DMSO) as solvent. The appropriate concentrations were obtained by diluting the stock solution in sterile distilled water, and the final concentration of DMSO in the incubation mixture never exceeded 0.1%. Control samples were always treated with the same amount of DMSO (0.1% v/v) as used in the corresponding experiments.

### Assays with mammalian cells

#### Culture conditions

The human cell lines MCF-7 (breast adenocarcinoma), HepG2 (hepatoma), T24 (bladder cancer), CACO-2 (colorectal adenocarcinoma) and MRC5 (lung normal fibroblast) were obtained from the Rio de Janeiro Cell Bank (Rio de Janeiro, RJ, Brazil). The HCT116 cell (colorectal carcinoma), and HT29 cell (colorectal adenocarcinoma) were kindly provided by Dr. Annette K. Larsen (Institut National de la Santé et de la Recherche Médicale - INSERM, Paris, França). All cell lines except MCF-7 were grown in DMEM supplemented with 10% or 20% (for CACO-2) FBS, 100 units.mL^-1^ penicillin and 100 μg.mL^-1^ streptomycin at 37°C in a humidified atmosphere of 5% CO_2_. MCF-7 cells were maintained in RPMI-1640 supplemented with 20% FBS at the same conditions described above.

### Cell viability assay

The cytotoxic potential of the TTHL was evaluated by the XTT assay in human tumor cell lines as well as in MRC5 normal fibroblasts. Cells (1 × 10^4^ cells) were seeded on 96-well plates in growth medium and incubated overnight. Afterwards, TTHL (0.5, 1.0, 2.5, 5.0, and 10 μg/mL) was added to each well and incubated for 24, 48, 72, and 120 h. Mitoxantrone, a cytostatic anthracenedione that intercalates in DNA and increases the incidence of double-strand breaks by stabilizing the cleavable complex of topoisomerase II and DNA, was used as positive control [18]. At the end of each treatment, cell viability was assessed according to the manufacturer’s instructions. Briefly, after discarding the medium, 1 mL of XTT labeling mixture was added to the cells and incubated for 2 h at 37°C. Absorbance was measured with a SpectraMax reader (Bio-Rad, USA) at a test wavelength of 492 nm (A_492_) and a reference wavelength of 690 nm (A_690_). The final result corresponds to A_492_-A_690_. The absorbance of negative control cells was set as 100% viability, and the values for treated cells were calculated as a percentage of the control.

All other experiments (comet, cell cycle, apoptosis, ROS production and protein expression assays) were conducted in the breast cancer cell line MCF-7 using IC20 = 0.50 μg/mL (20% inhibitory concentration), IC50 = 1.36 μg/mL (50% inhibitory concentration) and IC80 = 3.70 μg/mL (80% inhibitory concentration) values of TTHL, as determined by XTT assay. Cells (2 × 10^5^ cells/mL) were seeded on 6-well tissue culture plates, and grown for 1 day up to 70–80% confluence before treatment with the test substance. The TTHL was added to medium with FBS to obtain the different concentrations, and cells were treated at 37°C for 24 h in humidified atmosphere containing 5% CO_2_.

### Cell cycle distribution by flow cytometric analysis

Cell cycle distribution determination analysis was performed as previously described [19]. After treatment, cells were trypsinized, centrifuged and resuspended in ice-cold 70% ethanol, and left for 24 h at 4°C. Ethanol-fixed cells were centrifuged, washed with PBS and resuspended in buffer containing 0.2 mg/mL RNAse, 50 μg/mL PI and 0.1% Triton X-100, and incubated for 30 min at room temperature. Samples were analyzed on a FACS Calibur flow cytometer (Becton-Dikinson, San Fransisco, CA) using the CellQuest software. A total of 10,000 events were measured per sample. The data were analyzed to determine the percentage of cells at each phase of the cell cycle (sub-G1, G1, S and G2/M).

### Assessment of apoptosis by flow cytometric analysis

Annexin V-PE was used in conjunction with a vital dye, 7-AAD, to distinguish apoptotic (Annexin V-PE positive, 7-AAD negative) from necrotic (Annexin V-PE positive, 7-AAD positive) cells. After treatment, cells were trypsinized, collected and resuspended in 40 μL of binding buffer with 2 μL Annexin V-PE. Cells were incubated for 15 min in the dark at room temperature. After incubation, 160 μL of binding buffer and 2 μL of 7-AAD were added. Cells were incubated for 5 min and additional 200 μL of binding buffer were added. Before analysis, cells were filtered through a cell strainer cap fitted to a polystyrene round bottom flow cytometric tube. Data were collected and analyzed by a FACS Calibur flow cytometer with CellQuest software, in a total of 10,000 events per sample; fluorescence was measured and the percentage of viable, early apoptotic, late apoptotic and necrotic cells was determined.

### Quantification of cleaved caspase-9 by flow cytometric analysis

After treatment, cells (1 × 10^6^) were harvested, resuspended in 25 μL PBS and fixed with 4% formaldehyde. After permeabilization and blocking (0.2% Triton X-100 in PBS and 1% BSA), cells were incubated with anti-caspase-9 antibody (diluted 1:1000) for 1 h at room temperature, followed by incubation with anti-rabbit FITC secondary antibody (Uniscience) (diluted 1:1000) for 1 h at room temperature in the dark. A total of 10,000 events were analyzed per sample by FACS Calibur flow cytometer. Fluorescence intensity in arbitrary units was plotted in histograms; the mean fluorescence intensity was calculated using CellQuest software.

### Alkaline comet assay

The alkaline comet assay was performed as previously described [20]. Briefly, 10 μL of cell suspension (1 × 10^4^ cells) treated with TTHL were mixed with 90 μL LMP agarose, spread on a normal agarose precoated microscope slide, and placed at 4°C for 5 min to allow for solidification. Cells were lysed in high concentration of salt and detergent (2.5 M NaCl, 100 mM Na_2_EDTA, 10 mM Tris with 1% Triton X-100 and 10% DMSO freshly added) for 2 h. Slides were removed from lysing solution and washed three times with PBS. Subsequently, cells were exposed to alkali conditions (300 mM NaOH/1 mM Na_2_EDTA, pH >13, 30 min, 4°C) to allow DNA unwinding and expression of alkali-labile sites. Electrophoresis was conducted for 25 min at 25 V and 300 mA (94 V/cm). After electrophoresis, the slides were neutralized and silver stained [21]. One hundred cells were scored visually according to the tail length and the amount of DNA present in the tail. Each comet was given an arbitrary value of 0–4 (0, undamaged; 4, maximally damaged), as described by Collins *et al.* [22]. Damage score was thus assigned to each sample and can range from 0 (completely undamaged: 100 cells × 0) to 400 (with maximum damage: 100 cells × 4). International guidelines and recommendations for the comet assay consider that visual scoring of comets is a well-validated evaluation method, as it is highly correlated with computer-based image analysis [22, 23].

### Reactive oxygen species (ROS) detection by flow cytometric analysis

Levels of intracellular ROS were estimated following treatment with TTHL using 2′,7′-dichlorofluorescein diacetate (H_2_DCFDA, Sigma) as a fluorescent probe. Detection of oxidative stress was done by incubating the cells with 20 μM of H_2_DCFDA for 20 min at 37°C. Cells were then detached by trypsinization and washed twice with PBS. After filtration through cell strainer cap, cells were analyzed using a FACS Calibur flow cytometer with CellQuest software in accordance with Bass *et al.* [24]. A total of 10,000 events were measured per sample. DCF fluorescence intensity was shown in arbitrary units.

### Assays with S. cerevisiae

#### Strains, media and treatment

The relevant genotypes of yeast strains used in this work are shown in Table 1. Media, solutions and buffers were prepared according to Burke *et al.* [25]. YPD medium (0.5% yeast extract, 2% peptone, 2% glucose) was used for routine growth. Synthetic medium SC (0.67% yeast nitrogen base, 0.1% ammonium sulfate, 2% glucose) supplemented with the appropriate amino acids and bases (40 mg/mL) was used for the detection of mutations.

**Table 1:** ***Saccharomyces cerevisiae*** **strains used in this study**

Strain	Genotype	Enzymatic defense lacking	Source
EG103 (SOD-WT)	*MATα: leu2*Δ0 his 3-Δ1 trp1-289 ura 3-52	None	E. Gralla^a^
EG118 (*sod1*Δ)	*Like EG103, except sod1::URA3*	Cu-Zn SOD (cytosolic)	E. Gralla^a^
EG110 (*sod2*Δ)	*Like EG103, except sod2::TRP1*	Mn SOD (Mitochondrial)	E. Gralla^a^
EG133 (*sod1*Δ*sod2*Δ)	*Like EG103, except sod1::URA3 and sod2::TRP1*	Without SOD	E. Gralla^a^
XV185-14c (WT)	*MATα: ade2-2 his1-798 lys1-1 trp5-48 hom3-10 arg4-17*	None	von Borstel *et al.* (1971)^b^

^a^Department of Chemistry and Biochemistry, University of California, Los Angeles 90024–1569, USA. ^b^Von Borstel RC, Cain KT, Steinberg CM (1971) Inheritance of spontaneous mutability in yeast. Genetics 69:17–27.

Stationary phase (STAT) cultures were obtained by inoculation of a single colony into liquid YPD. We chose to work in the stationary phase of growth because this resembles most cells of multicellular organisms in important aspects: (i) most energy comes from mitochondrial respiration; (ii) the cells have left the active cell cycle and have entered the Go phase; and (iii) damage accumulates over time [26, 27].

### Survival assays in the EG103 background strains

STAT cells (1 × 10^8^ cells/mL) were exposed to TTHL (10–500 μg/mL) and incubated under growth conditions for 1 h in PBS at 30°C. Cells were then washed and treated with H_2_O_2_ (5 mM) in PBS for another hour. Suitable aliquots were plated in triplicate on solid YPD (2–3 days, 30°C) and colony-forming units were counted. Sensitivity was expressed as a percentage of survival in relation to the negative control (solvent) [28].

### Point and frameshift mutations in the XV185-14c haploid yeast

Cell cultures were grown as described above, exposed to TTHL in concentrations ranging from 10 to 500 μg/mL, and then incubated in PBS for 1 h at 30°C. Two alleles, *his1-798* and *lys1-1*, were used to detect point mutagenesis. The suppressible ochre nonsense mutant allele *lys1-1* can be reverted either by locus-specific sequence alteration (true reversion) or by a forward mutation in a suppressor gene. Distinction between true reversions and forward (suppressor) mutations at the *lys1-1* locus was performed according to Schuller & Von Borstel [29], where the reduced adenine content of the SC-lys medium shows true reversions as red and suppressor mutations as white colonies. Survival was determined on SC medium (3–5 days, 30°C) and mutation induction (HIS, LYS or HOM revertants) on media lacking the appropriate amino acid (7–10 days, 30°C). Induction of reversion of point mutation to *his1-798*, ochre allele *lys1-1*, and frameshift mutation values were scored per number of surviving cells.

### Statistical analysis

The IC20, IC50, and IC80 values and their 95% confidence intervals (CI 95%) were obtained by nonlinear regression using GraphPad Prism v5 program (Intuitive Software for Science, San Diego, CA, USA). Selective index (SI) was calculated by IC50 in MRC5 cells/IC50 in tumoral cells. All experiments were independently repeated at least three times, with triplicate samples for each treatment. Results are expressed as means ± standard deviation (SD). Data were analyzed by one-way analysis of variance (ANOVA), and means were compared using Tukey test, with P ≤ 0.05 considered as statistically significant.

## Results

### Effects of TTHL on tumoral cells

Table 2 shows the cytotoxic effects of TTHL on cell lines. The antiproliferative effects were quantified in terms of IC50 values. The lower the IC50 value, the higher the antiproliferative activity. TTHL induced concentration-dependent cytotoxic effects in all the examined cell lines after 24, 48, 72 and 120 h. The ratio between the cytotoxic parameters found in MRC5 cells and those observed in tumoral cell lines can be considered as a measurement of compound selectivity index (SI). SI ratios between 3 and 6 refer to moderate selectivity, and ratios higher than six indicate high selectivity, whereas compounds that do not fulfill either of these criteria are rated as non-selective [30, 31]. The SI value is also shown in Table 2 (superscript).

**Table 2:** **Antiproliferative activity of TTHL on human cell lines**

	IC50 (μg/mL) ^a^ ± SD						
Time of Treatment (h)	Selectivity index (SI) ^b^/superscript value						
	MRC5	MCF-7	HepG2	T24	HCT116	HT29	CACO-2
**24**	8.40 ± 0.28	1.36 ± 0.05^**6X**^	6.50 ± 0.43^**1.3X**^	5.55 ± 0.64^**1.5X**^	7.05 ± 0.21^**1X**^	8.00 ± 0.14^**1X**^	8.55 ± 0.35^**MR**^
**48**	7.60 ± 0.14	0.73 ± 0.05^**10X**^	6.10 ± 0.28^**1X**^	5.20 ± 0.42^**1.5X**^	5.87 ± 0.04^**1X**^	7.00 ± 0.42^**1X**^	7.90 ± 0.28^**MR**^
**72**	7.40 ± 0.15	0.63 ± 0.04^**12X**^	5.45 ± 0.35^**1X**^	1.09 ± 0.14^**7X**^	5.20 ± 0.28^**1X**^	6.15 ± 0.21^**1X**^	6.35 ± 0.35^**1X**^
**120**	6.84 ± 0.10	0.30 ± 0.04^**23X**^	0.88 ± 0.13^**8X**^	0.39 ± 0.10^**17X**^	4.50 ± 0.29^**1.5X**^	5.30 ± 0.28^**1X**^	5.00 ± 0.19^**1X**^
**24/MXT** ^**c**^	2.88 ± 1.44	0.87 ± 1.59	3.5 ± 0.39	2.50 ± 0.62	0.61 ± 0.67	0.88 ± 2.33	2.4 ± 1.81

^a^Drug concentration required to inhibit the cell growth by 50% after 24 h of incubation. ^b^Selectivity index (*in vitro*): IC50 in MRC5 cells/IC50 in tumoral cells. Data represent mean ± three separate experiments. ^c^Mitoxantrone (MXT) was used as positive control. MR: more resistant in tumoral cells.

Interestingly, our results suggest that TTHL exerted the highest cytotoxicity against MCF-7 cells, with an IC50 value of 1.36 ± 0.05 after 24 h of treatment and a higher potency - six fold more potent in these cells than in normal cells (MRC5). In addition, TTHL also showed a significant inhibitory activity in human cancer cell line T24, but only after 72 h of treatment (Table 2). Comparatively, mitoxantrone, used in this study as a positive control, demonstrated IC50 values ranging from 0.61-3.5 μg/mL in tumor cell lines. Considering both cytotoxic parameters and selectivity index, TTHL displayed the best profile in MCF-7 cells. Therefore, we chose the MCF-7 cells for further experiments to verify the cytotoxic mechanism of TTHL, using concentrations of 0.50 μg/mL, 1.36 μg/mL and 3.70 μg/mL (IC20, IC50 and IC80, respectively).

### Cell cycle distribution by flow cytometric analysis

**Figure 2:**
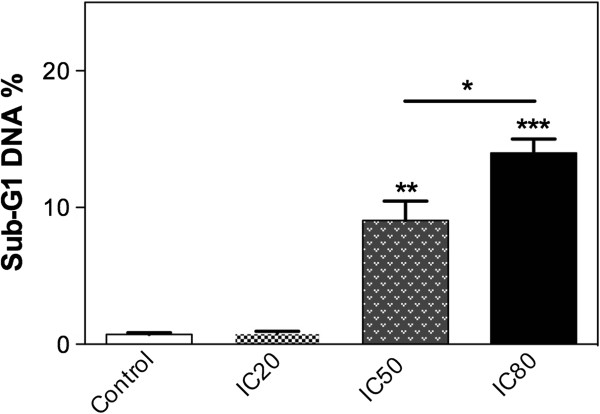
**The subdiploid G1 peak from FACS analysis was used to estimate the apoptotic cell population on MCF-7 cells after 24 hours’ treatment.** Sub-G1 populations are seen to appear at both IC50 and IC80. Statistical analysis was applied for comparing control non-treated cells with treated cells. Data are expressed as mean ± SD of three independent experiments.

The results showed there was no significant change in cell cycle progression after treatment with TTHL (Additional file 1: Figure S8); however, a significant increase in the sub-G1 population can be observed (Figure 2). It is important to note that flow cytometry after PI staining allows cell cycle analysis and, additionally, any cells undergoing apoptosis can be detected as a subdiploid peak [19]. The increased number of cells in sub-G1 phase population appears to be concentration-dependent (Figure 2), indicating that TTHL may induce cell death by apoptosis.

### Apoptosis by flow cytometric analysis

**Figure 3:**
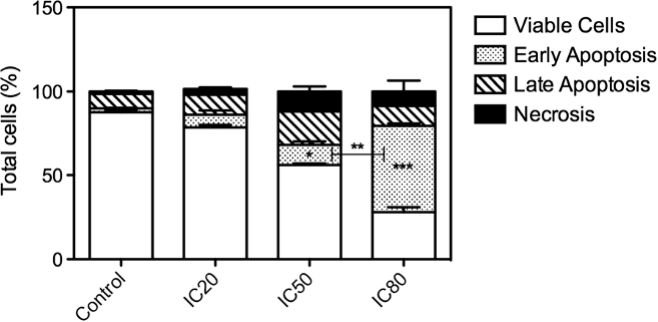
**TTHL treatment induces apoptosis in MCF-7 cells.** Annexin V assays analyzing in MCF-7 cells treated with IC20, IC50, and IC80 TTHL. The sum of the percentages of Annexin V and 7-AAD-PE-positive cells was calculated. Three independent experiments were pooled and analyzed as a combined data set. Results are expressed as means ± standard deviation (SD). Data were analyzed by one-way analysis of variance (ANOVA), and means were compared using Tukey test, with P ≤ 0.05 considered as statistically significant.

The morphology of treated MCF-7 cells was analyzed using annexin V-PE/7-AAD staining and the percentages of viable, apoptotic and necrotic cells were calculated. After 24 h of TTHL (IC80) treatment, approximately 55% MCF-7 cells were in early apoptosis (labeled with annexin V-PE), 15% cells were in late apoptosis (labeled with both annexin V-PE and 7-AAD), and a remarkable decrease was observed in the viable cells population (unlabeled with either annexin-V or 7-AAD) compared with the untreated control cells (Figure 3, and Additional file 2: Figure S9). The percentage of necrotic cells (labeled with 7-AAD) was less than 5% for both TTHL treatments (IC50 and IC80), and the dominant cell death type was apoptosis.

### Quantification of cleaved caspase-9 by flow cytometric analysis

**Figure 4:**
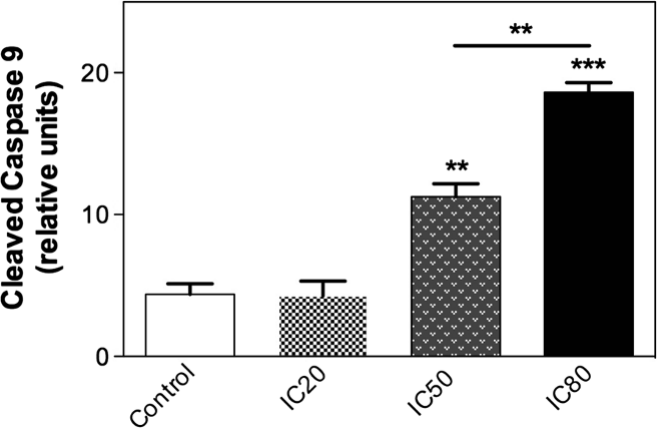
**Flow cytometric analysis of cleaved caspase 9 activity.** MCF-7 cells were treated with TTHL (IC20 = _0.50_ μg/mL, IC50 = _1.36_ μg/mL, and IC80 = _3.70_ μg/mL), fixed, permeabilized, and stained with anti-active caspase 9. Results are expressed as means ± standard deviation (SD). Data were analyzed by one-way analysis of variance (ANOVA), and means were compared using Tukey test, with P ≤ 0.05 considered as statistically significant.

In order to characterize the apoptosis induced in MCF-7 cell line, we evaluated the activation of apoptosis mediators by different TTHL concentrations. We analyzed the cleavage and consequent activation of caspase-9 by flow cytometry using specific antibodies that recognize only the intact (inactive) forms of the enzyme. The results, summarized in Figure 4, indicate that continuous treatment of cells with TTHL concentrations (IC50 and IC80) leads to increased induction of cleaved caspase-9-labeled cells. There is a clear difference on the induction of cleaved caspase-9 when the IC50 and IC80 values are compare (Figure 4), although the increased induction of cell death (sub-G1) in treated MCF-7 cells is confirmed (Figure 2, and Additional file 1: Figure S8). Moreover, TTHL (at IC20) caused no significant changes in the levels of cleaved caspase-9 in MCF-7 cells (Figure 4). These results were consistent with the data from Annexin V-PE positive FACS analysis; in fact, the treatment with TTHL at IC50 and IC80 induced a stronger apoptotic effect on MCF-7 cells when compared to IC20 and control (Figures 2, 3 and 4).

### Comet assay

**Figure 5:**
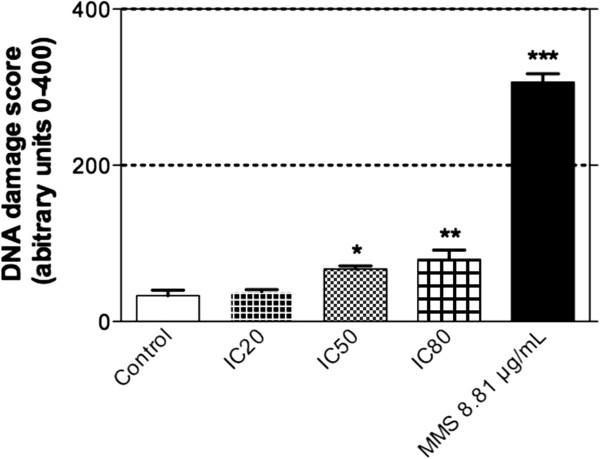
**Effect of TTHL after 24 h of treatment on DNA damage index using MCF-7 cells, determined by comet assay.** Control (untreated) or treated cells with TTHL at concentrations IC20, IC50, and IC80 were used. Data are expressed as mean ± SD of three independent experiments.

The alkaline (pH > 13) comet assay detects DNA strand breaks and alkali-labile sites. Our results showed that TTHL does not generate DNA-strand breaks at IC20. However, it induces signifcant DNA damage at IC50 and IC80 (Figure 5). MMS (8.81 μg/mL) was used as positive control.

### Intracellular increase of ROS is responsible for apoptosis induced by TTHL in MCF-7 cells

**Figure 6:**
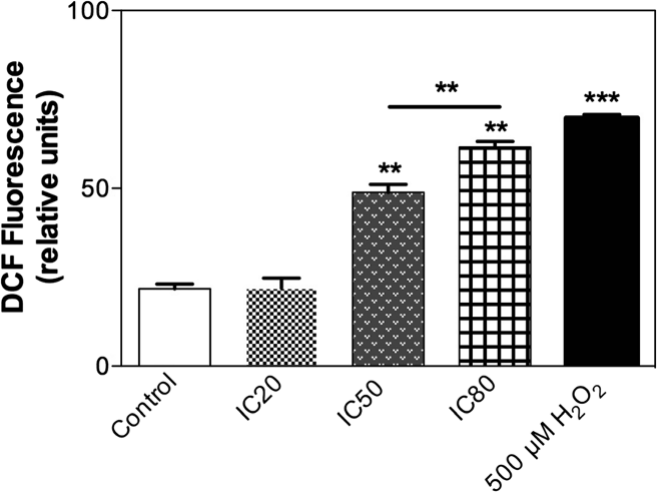
**Flow cytometry detection of reactive oxygen species in MCF-7 cells challenged with TTHL.** Cells were treated with vehicle negative control, IC20 = _0.50_ μg/mL, IC50 = _1.36_ μg/mL, and IC80 = _3.70_ μg/mL TTHL after 24 hours’s treatment, and 500 μM H_2_O_2_. Mean data for DCF fluorescence. Results are expressed as means ± standard deviation (SD). Data were analyzed by one-way analysis of variance (ANOVA), and means were compared using Tukey test, with P ≤ 0.05 considered as statistically significant.

Results shown in Figure 6 and Additional file 3: Figure S10 revealed that TTHL induces an increase in intracellular ROS production (24 h treatment at IC50: 49% increase; 24 h treatment at IC80: 64% increase) in MCF-7 cells, as measured by flow cytometry.

### Effects of TTHL on yeast cells

#### Cytotoxic and mutagenic effects in S. cerevisiae

TTHL exhibited a concentration-dependent cytotoxic effects in all strains, with more pronounced effect in double mutant *sod1*Δ*sod2*Δ (lacks cytoplasmic and mitochondrial superoxide dismutase enzymes) (Table 3). The mutagenesis induction was evaluated and the results showed that TTHL was cytotoxic in XV185-14c in all concentrations tested. Notwithstanding, TTHL was only able to induce mutagenesis on the assessed loci at the highest concentration (Table 4).

**Table 3:** **Cytotoxicity and antioxidant effect of TTHL isolated product from ethnolic extract of** ***C. leprosum*** **in** ***S. cerevisiae***

Yeast strains				
Treatment	***WT***	***sod1***Δ	***sod2***Δ	***sod1***Δ***sod2***Δ
NC^a^	100.0 ± 0.0	100.0 ± 0.0	100.0 ± 0.0	100.0 ± 0.0
TTHL 10 μg/mL	100 ± 2.83	92.60 ± 2.83	83.20 ± 3.68**	79.35 ± 2.19**
TTHL 50 μg/mL	87.65 ± 5.16**	89.30 ± 0.85**	72.90 ± 7.07***	67.85 ± 10.54***
TTHL 100 μg/mL	79.45 ± 1.91***	75.65 ± 4.60***	62.25 ± 3.32***	58.15 ± 3.89***
TTHL 500 μg/mL	67.65 ± 3.04***	69.30 ± 3.68***	56.65 ± 1.06***	41.45 ± 4.03***
PC^b^: H_2_O_2_ 5 mM	63.03 ± 4.12	16.27 ± 4.88	19.10 ± 4.03	19.33 ± 2.10

^a^NC: negative control (solvent-DMSO). Data significant in relation to negative control group (solvent) at *P < 0.05, **P < 0.01 and ***P < 0.001/ one-way ANOVA Tukey’s multiple comparison test. ^b^Positive control (H_2_O_2_).

**Table 4:** **Induction of reversion of point mutation for** ***his1-798*** **, ochre allele** ***lys1-1*** **, and** ***frameshift*** **mutation (** ***hom3-10*** **) in haploid strain XV185-14c of** ***S. cerevisiae*** **after treatment of TTHL isolated product from ethanolic extract of** ***C. leprosum***

Agent	Treatment (μg/mL)	Survival (%)	LYS1/10 ^8^survivors ^b^	HIS1/10 ^7^survivors ^a^	HOM3/10 ^8^survivors ^a^
STAT cells treated in PBS					
NC^d^	0	100.00	4.00 ± 2.83^c^	10.50 ± 0.71^c^	4.0 ± 1.41^c^
4-NQO^e^	1.0 μg/mL	39.97***	20.85 ± 2.48***	49.50 ± 9.19***	13.50 ± 4.95**
**TTHL**	10 μg/mL	84.90	4.78 ± 0.63	10.77 ± 4.40	6.11 ± 1.58
	50 μg/mL	72.83	5.68 ± 2.22	13.33 ± 1.10	8.80 ± 0.14
	100 μg/mL	65.00	9.72 ± 0.55	20.83 ± 1.80	8.49 ± 2.12
	500 μg/mL	69.00	14.40 ± 3.40**	30.10 ± 0.21**	11.15 ± 0.92*

^a^Locus-specific revertants; ^b^Locus non-specific revertants; ^c^Mean and standard deviation per three independent experiments; ^d^Negative control (solvent); ^e^Positive control; *Data significant in relation to negative control group (solvent) at *P < 0.05; **P < 0.01; ***P < 0.001/ One-way ANOVA-Tukey’s Multiple Comparison Test.

## Discussion

Drug discovery from medicinal plants has played an especially important role in the treatment of cancer and, indeed, over the last half century, most new clinical applications of plant secondary metabolites and their derivatives have been applied towards combating cancer [32]. Of all the available anticancer drugs between 1940 and 2002, 40% were natural products or natural product-derived, with another 8% considered natural product mimics [33–35]. Anticancer agents from plants currently in clinical use can be categorized into four main classes of compounds: vinca (or *Catharanthus*) alkaloids, epipodophyllotoxins, taxanes, and camptothecins [32].

Alternatively, the pentacyclic triterpenes are a group of promising secondary plant metabolites for cancer treatment. The triterpenes belonging to the lupane, oleanane or ursane groups have the potential to treat cancer by different mechanisms of action [32]. Since Pisha *et al.* [36] reported that betulinic acid is a highly promising anticancer drug, inducing apoptosis in melanoma cell lines *in vitro and in vivo*, experimental work has focused on the apoptosis-inducing mechanisms.

Accumulating data indicate that the cytotoxic effect of many chemotherapeutic drugs occurs through programmed cell death (apoptosis) [37, 38]. Hence, the ability of tumor cells to respond and activate the apoptotic program may, in part, determine the success of the therapeutic strategy [39]. It is well documented that apoptosis can be induced by a variety of drugs with diverse chemical structures and different mechanisms of action, and two major routes including the death-receptor pathway and the mitochondrial-pathway have been identified [40]. Apoptosis is a highly regulated process that involves many proteins and genes [40, 41]. It is characterized by cell shrinkage, plasma membrane bebbling, and chromatin condensation. The death program is executed by caspases, which amplify the apoptotic signal and proteolytically process numerous cellular molecules with different functions [40–42].

Caspases, a family of cysteine proteases, are central components of cellular apoptosis [41, 42]. Based on their different functions and structures, caspases are classified into two groups. Caspase-1, -2, -8, -9, and -10 belong to the first group, called initiator caspases, which can autocleave, and activate the second group of caspases, referred to as executioner caspases, which include caspase-3, -6, and -7 [42]. Accumulating evidence reveals that inhibition of apoptosis cascades plays an important role in tumor therapy resistance. For example, up-regulation of caspase-8 inhibitors like Flice-like inhibitory protein or inhibition of caspase-8 by Bcl-2 can induce tumor resistance to chemotherapy drugs by decreasing cellular apoptosis [43]. Suppressing the activation of caspase-9 downstream can cause chemotherapy resistance in diffuse large B-cell lymphoma [44]; overexpression of the inhibitor of caspase-3 can activate deoxyribonuclease in human renal carcinoma cells, therefore enhancing their resistance to cytotoxic chemotherapy [45]. These studies strongly suggest that regulating the activity of caspases might be beneficial in tumor chemotherapy.

In order to evaluate the new compound TTHL, obtained from *C. Leprosum*, a folklore medicinal plant, as a potential therapy for cancer, different concentrations and exposure times were studied against a panel of six human tumoral cell lines: MCF-7, HepG2, T24, HCT116, HT29, CACO-2 (Table 2). We showed that TTHL induces an increase in sub-G1 population (Figure 2), and the induction of apoptosis by TTHL was confirmed by the increasing population labeled with Annexin V (Figure 3). We also observed that there is an increase in the cleavage of caspase-9, a mediator of the execution phase of apoptosis. This suggests a mytochondria-mediated triggering of the apoptotic program in MCF-7 cells that is consistent with the involvement of oxidative stress (Figure 6 and Table 3).

Here we found that TTHL treatment induced an increase in intracelular ROS formation in MCF-7 cell, as measured by DCF oxidation (Figure 6). Corroborationg with these results, TTHL also induced sensitivity in strains without superoxide dismutase enzyme (Table 3), which reinforced the indirect action caused by TTHL through ROS generation in both yeast and mammalian cells. This suggests that the cellular toxicity of TTHL must be related with its capacity of TTHL to produce ROS, and the electrophilicity of TTHL, which enables to form adducts with cellular macromolecules.

Subsequent experiments focused on direct interaction of TTHL with DNA. Chemical compounds bearing planar topologies and electrophilicity are often capable of intercalating between DNA bases [46]. As TTHL is an electrophilic molecule with a polar group at either 3-OH, 6-OH, or 16-OH positions, and was capable of inducing frameshift mutation (Table 4), we suggest TTHL is a weak intercalator mutagen.

In addition to the mutagenicity assay, the comet assay is a sensitive and valuable technique to observe genotoxic damage. The comet assay detects primary (repairable) single and double-strand DNA breaks and alkali-labile sites in the alkaline test version. The comet assay results indicate that TTHL induced DNA strand breaks in MCF-7 cells (Figure 5). Thus, the observed increase in DNA migration in comet assay could be explained by either base damage or strand breaks induction by ROS generation or by direct action of TTHL on DNA. In this way, the TTHL DNA-damaging effect occurs not only by ROS generation but also via DNA intercalation (Table 4).

It is known that various triterpenoids are able to intervene in such processes as DNA repair, cell proliferation, cell differentiation, angiogenesis and apoptosis [32]. The antitumoral effects of ursolic acid, maslinic acid and betulinic acid have been reported to stimulate similar apoptotic mechanisms. Ursolic acid induces apoptosis via the mitochondrial intrinsic pathway with alterations of the Bax/Bcl-2 balance in M4Beu cells [47] and its anti-carcinogenic properties have also been described [48]. Several attempts were undertaken for the derivatization of ursolic acid, seeking to obtain analogs with improved anti-tumor activity [32]. Ma et al. [49] modified the C-3, C-28, C-11 positions of ursolic acid and, among the 23 derivatives they synthesized, 3β-amino derivative was found to be 20 times more potent than the parent ursolic acid in HL-60, Bel-7402 and HeLa cell lines. Usually, compounds with β-oriented hydrogen-bond forming groups at C-3 exhibit more potent cytotoxicity than their α-counterparts [32]. Analyzing the structure-activity relationship (SAR), it appears that a polar group at either 3-OH, 6-OH, or 16-OH positions is essential for the cytotoxic activity of TTHL (Figure 1 and Table 1). Especially, TTHL showed higher activity in antiproliferation assays in MCF-7 cells.

More recently, some studies have shown that maslinic acid, a natural pentacyclic triterpene has anti-cancer capacity in different cell types, including melanoma [50], liver cancer [51], astrocytoma [52], and colon cancer [53]. Specifically in colon malignancies, maslinic acid possesses potent differentiating and anti-proliferation properties, inducing cell-cycle arrest in the G0/G1 phase and apoptosis in colon cancer cells without affecting non-tumoral cells [53].

The induction of apoptosis by betulinic acid involves several mitochondrial perturbations, such as the release of cytochrome-c and activation of caspase-8 [54]. Furthermore, it has been reported that the protein Smac is released during the induction of apoptosis by betulinic acid, whereas with other tumoral cells it provokes the down-regulation of Bcl-2, thus blocking the release of anti-apoptogenic molecules [55]. In addition, the formation of ROS that modulate Bcl-2 and Bax levels during the action of betulinic acid has also been detected [56].

Other terpenoids, such as amooranin, induce apoptosis in MDA-468 cells via the activation of caspases -9, -3, and -8, the cleavage of Bid and the release of cytochrome-c from the mitochondria, concomitant with the up-regulation of p53 and Bax and down-regulation of Bcl-2 [57]. In addition, an increase in the Bax/Bcl-2 ratio and a decrease in mitochondrial membrane potential have been reported as being involved in the induction of apoptosis for alisol B acetate [58].

## Conclusions

**Figure 7:**
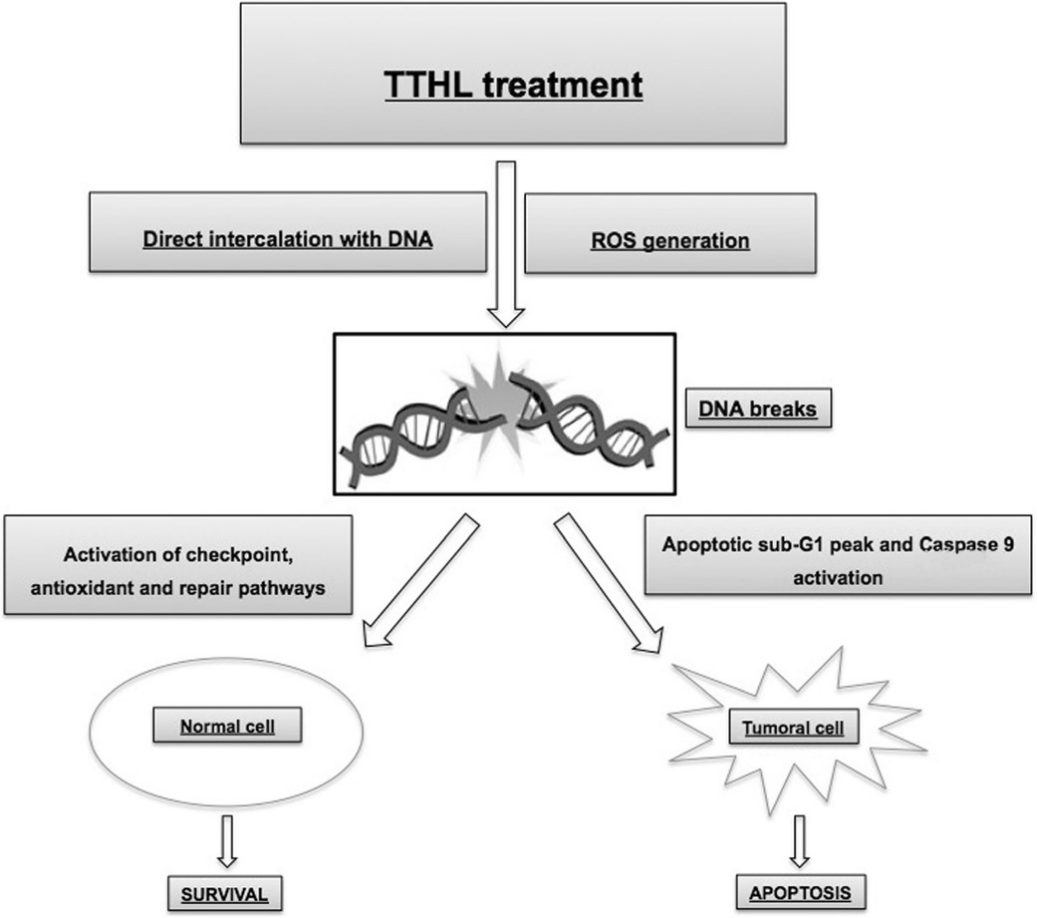
**Schematic representation of the plausible molecular mechanism proposed for the induction of apoptosis by TTHL in MCF-7 breast cancer cells.** ROS overproduction and DNA intercalaiton lead to DNA damage and mitochondrial dysfunction, resulting in the release of mitochondrial pro-death proteins. There is an incresead in the number of cells within the sub-G1 phase with a maximum at IC80. The intrinsic apoptosis pathway is initiated by lose of mitochondrial potential, which leads to release of cytochrome c. Cytochrome c binds to APAF-1 and procaspase 9, resulting in activation of initiator caspase 9 and downstream executor caspases.

In this paper the ROS formation by TTHL and its direct interaction with DNA are presented. As indicated in Figure 7, treating MCF-7 cells with TTHL causes cascade signaling in the induction of caspases, which in turn governs the mechanisms for inducing apoptosis. Initiator caspase-9 was activated significantly after 24 h. The apoptotic potency of TTHL suggests that it may be an effective compound in breast cancer therapy. Further studies with other biological models are currently being performed in our laboratory and should provide a better understanding of the mechanisms underlying these effects.

This is one of the first studies demonstrating the *in vitro* antitumor activity of TTHL in MCF-7 cells. The antitumor effect of TTHL can be further enhanced by the use of combined therapy and novel drug delivery systems, thus making it a promising candidate for management of breast cancer patients.

## Electronic supplementary material

Additional file 1: Figure S8: Cell cycle profile of MCF-7 cells after 24 hours’ treatment with TTHL (IC20, IC50, and IC80) for 24 h. A hypodiploid peak can be seen in the sub-G1 region. Sub-G1 populations are seen to appear at both IC50 and IC80. Blue line: control, Red line: IC20, Green line: IC50, and Pink line: IC80. (JPEG 20 KB)

Additional file 2: Figure S9: Apoptosis induction in MCF-7 cells. TTHL-induced apoptosis as shown in the representative example of Annexin V flow cytometry analysis with the x axis showing Annexin V staining and the y axis 7-amino-actinomycin D (7-AAD) and phycoerythrin (PE) staining. The percentage of annexin-V-positive cells was determined in the whole-cell population (10,000 cells) by FACSCalibur flow cytometry and CELLQuest software. Percentage of cells in each quadrant, LL: Viable cells (Annexin V -/ PE -), LR: early apoptotic cells (Annexin V +/ PE -), UL: necrotic cells (Annexin V -/ PE +) and UR: late apoptotic/necrotic cells (Annexin V +/ PE +). (JPEG 59 KB)

Additional file 3: Figure S10: Flow cytometry detection of reactive oxygen species in MCF-7 cells challenged with TTHL. (A) Representative histograms: number of cellular events versus fluorescence intensity. FL1-H: relative DCF fluorescence intensity. Cells were treated with vehicle negative control, IC20 = _0.50_ μg/mL, IC50 = _1.36_ μg/mL, and IC80 = _3.70_ μg/mL TTHL after 24 hours’s treatment, and 500 μM H_2_O_2_. (JPEG 49 KB)
