# Supplementary figures and images for: The natural triterpene 3β,6β,16β-trihydroxy-lup-20(29)-ene obtained from the flowers of Combretum leprosum induces apoptosis in MCF-7 breast cancer cells
Source: BMC Complement Altern Med. 2014 Aug 2;14:280. doi: 10.1186/1472-6882-14-280 (PMC4129108; doi:10.1186/1472-6882-14-280)

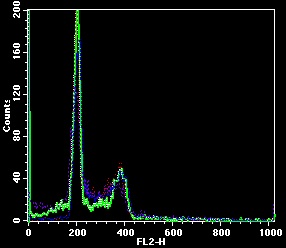

Supplement: Supplementary file 1 — Additional file 1: Figure S8: Cell cycle profile of MCF-7 cells after 24 hours’ treatment with TTHL (IC20, IC50, and IC80) for 24 h. A hypodiploid peak can be seen in the sub-G1 region. Sub-G1 populations are seen to appear at both IC50 and IC80. Blue line: control, Red line: IC20, Green line: IC50, and Pink line: IC80. (JPEG 20 KB) [file 12906_2014_1857_MOESM1_ESM.jpeg]

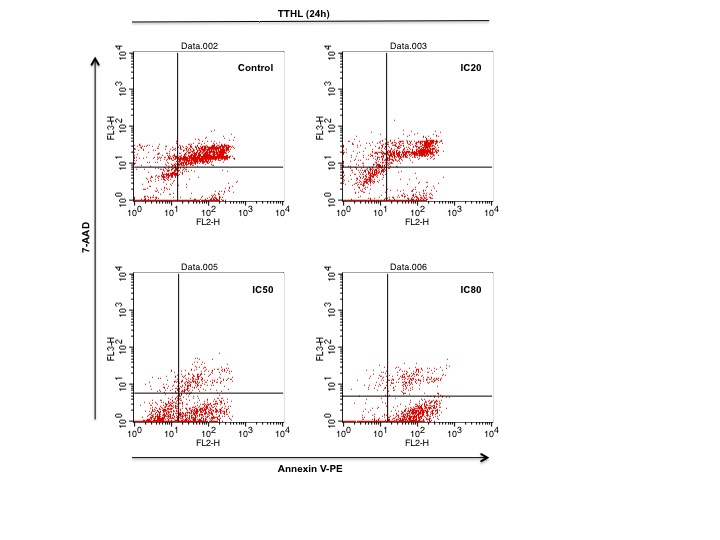

Supplement: Supplementary file 2 — Additional file 2: Figure S9: Apoptosis induction in MCF-7 cells. TTHL-induced apoptosis as shown in the representative example of Annexin V flow cytometry analysis with the x axis showing Annexin V staining and the y axis 7-amino-actinomycin D (7-AAD) and phycoerythrin (PE) staining. The percentage of annexin-V-positive cells was determined in the whole-cell population (10,000 cells) by FACSCalibur flow cytometry and CELLQuest software. Percentage of cells in each quadrant, LL: Viable cells (Annexin V -/ PE -), LR: early apoptotic cells (Annexin V +/ PE -), UL: necrotic cells (Annexin V -/ PE +) and UR: late apoptotic/necrotic cells (Annexin V +/ PE +). (JPEG 59 KB) [file 12906_2014_1857_MOESM2_ESM.jpeg]

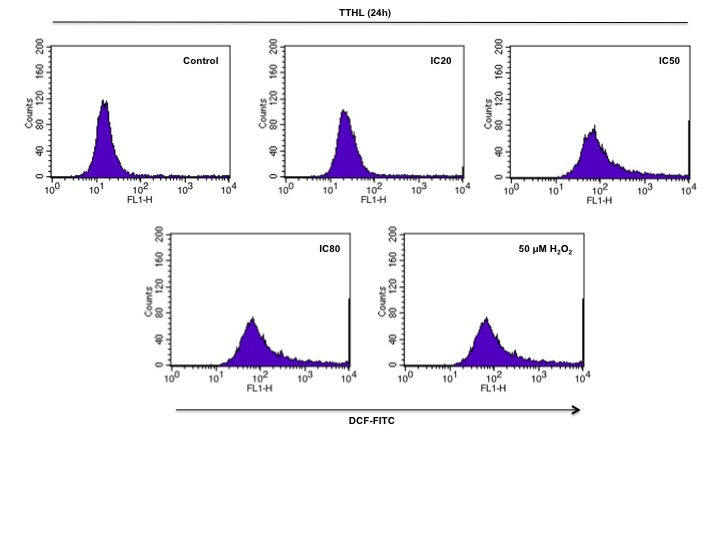

Supplement: Supplementary file 3 — Additional file 3: Figure S10: Flow cytometry detection of reactive oxygen species in MCF-7 cells challenged with TTHL. (A) Representative histograms: number of cellular events versus fluorescence intensity. FL1-H: relative DCF fluorescence intensity. Cells were treated with vehicle negative control, IC20 = 0.50 μg/mL, IC50 = 1.36 μg/mL, and IC80 = 3.70 μg/mL TTHL after 24 hours’s treatment, and 500 μM H2O2. (JPEG 49 KB) [file 12906_2014_1857_MOESM3_ESM.jpeg]
